# Mannose-Specific Lectins from Marine Algae: Diverse Structural Scaffolds Associated to Common Virucidal and Anti-Cancer Properties

**DOI:** 10.3390/md17080440

**Published:** 2019-07-26

**Authors:** Annick Barre, Mathias Simplicien, Hervé Benoist, Els J.M. Van Damme, Pierre Rougé

**Affiliations:** 1 Institut de Recherche et Développement, Faculté de Pharmacie, UMR 152 PharmaDev, Université Paul Sabatier, 35 Chemin des Maraîchers, 31062 Toulouse, France; 2 Department of Biotechnology, Faculty of Bioscience Engineering, Ghent University, Coupure links 653, B-9000 Ghent, Belgium

**Keywords:** lectin, seaweed, red algae, mannose-binding specificity, structure-function relationships, diagnostic tool, therapeutic drugs, anti-cancer properties, anti-HIV-1 properties

## Abstract

To date, a number of mannose-specific lectins have been isolated and characterized from seaweeds, especially from red algae. In fact, man-specific seaweed lectins consist of different structural scaffolds harboring a single or a few carbohydrate-binding sites which specifically recognize mannose-containing glycans. Depending on the structural scaffold, man-specific seaweed lectins belong to five distinct structurally-related lectin families, namely (1) the griffithsin lectin family (β-prism I scaffold); (2) the *Oscillatoria agardhii* agglutinin homolog (OAAH) lectin family (β-barrel scaffold); (3) the legume lectin-like lectin family (β-sandwich scaffold); (4) the *Galanthus nivalis* agglutinin (GNA)-like lectin family (β-prism II scaffold); and, (5) the MFP2-like lectin family (MFP2-like scaffold). Another algal lectin from *Ulva pertusa*, has been inferred to the methanol dehydrogenase related lectin family, because it displays a rather different GlcNAc-specificity. In spite of these structural discrepancies, all members from the five lectin families share a common ability to specifically recognize man-containing glycans and, especially, high-mannose type glycans. Because of their mannose-binding specificity, these lectins have been used as valuable tools for deciphering and characterizing the complex mannose-containing glycans from the glycocalyx covering both normal and transformed cells, and as diagnostic tools and therapeutic drugs that specifically recognize the altered high-mannose *N*-glycans occurring at the surface of various cancer cells. In addition to these anti-cancer properties, man-specific seaweed lectins have been widely used as potent human immunodeficiency virus (HIV-1)-inactivating proteins, due to their capacity to specifically interact with the envelope glycoprotein gp120 and prevent the virion infectivity of HIV-1 towards the host CD4+ T-lymphocyte cells in vitro.

## 1. Introduction

Lectins have been identified as sugar-binding proteins, in all groups of living organisms including plants, animals, fungi and bacteria, and even in viruses and mycoplasms. Depending on their broad sugar-binding specificity, they have been classified as mannose-, galactose-, *N*-acetyl-glucosamine-, fucose- and sialic acid-binding lectins, according to the simple sugars that inhibit their carbohydrate-binding properties. In fact, only a few lectins are not inhibited by simple sugars since they only recognize complex glycan chains [1]. Another classification system of lectins in twelve different families is based on the structural organization of the lectin domains, irrespective of their sugar-binding specificities [2].

Compared to other plant and animal man-specific lectins, not much attention has been paid to man-specific seaweed lectins, with the exception of griffithsin, a man-specific lectin isolated from the red alga *Griffithsia* sp. [3], owing to its outstanding anti-human immunodeficiency virus (HIV) properties. Like other man-specific lectins, griffithsin specifically recognize some of the high-mannose *N*-glycans exposed at the surface of gp120, thus interfering with the recognition of gp120 by the CD4+ T lymphocytes. In fact, the association of three gp120-gp41 tandems forms the HIV-1-envelope spike, which facilitates HIV-1 entry into cells. The Env spike of HIV-1 consists of a transmembrane trimer of gp41 associated to an extracellular trimer of gp120, offering exposed high-mannose glycans to the CD4 recognition process (see Section 6.1). In addition to griffithsin, other man-specific lectins belonging to different groups of algae including Rhodophyta (red algae), Pheophyceae (brown algae), Xanthophyceae (yellow-green algae), and Chlorophyta (green algae), have been identified and characterized, particularly with respect to their anti-HIV properties [4,5]. In addition to their affinity for high-mannose glycans, both cloning and structural information have become available for a few seaweed lectins.

The present review presents an exhaustive overview on the structure-function relationships of man-specific lectins from seaweeds, particularly in relation with their potential biomedical applications as anti-HIV and anticancer agents.

## 2. Diversity of Mannose-Binding Lectins in Seaweeds

Mannose-specific lectins have been identified in almost all the different families of higher plants belonging to monocot and dicot groups [6]. In addition to higher plant lectins, other man-specific lectins were found in lower plant species, algae and mushrooms [7]. To date, however, a still discrete number of alga species were successfully investigated with the aim of identifying and characterizing man-specific lectins, susceptible to be used as relevant biomarkers for HIV gp120 and cancer high-mannose glycoforms. In this respect, red algae or Rhodophyta, appear as the most promising source for man-specific lectins (Table 1).

## 3. Structural Scaffolds of the Man-Specific Seaweed Lectins

Mannose-specific lectins from seaweeds essentially belong to four distinct structural scaffolds corresponding to monomeric structures that occur as single carbohydrate-binding modules or subsequently arrange in homodimeric structures. In addition, complex proteins with different functions occurring in seaweeds contain a lectin domain corresponding to a carbohydrate-binding module (CBM) with man-specificity, e.g., the legume-lectin like domain in the BU14 protein of *Porphyra umbilicalis* [20].

### 3.1. Griffithsin

**Figure 1:**
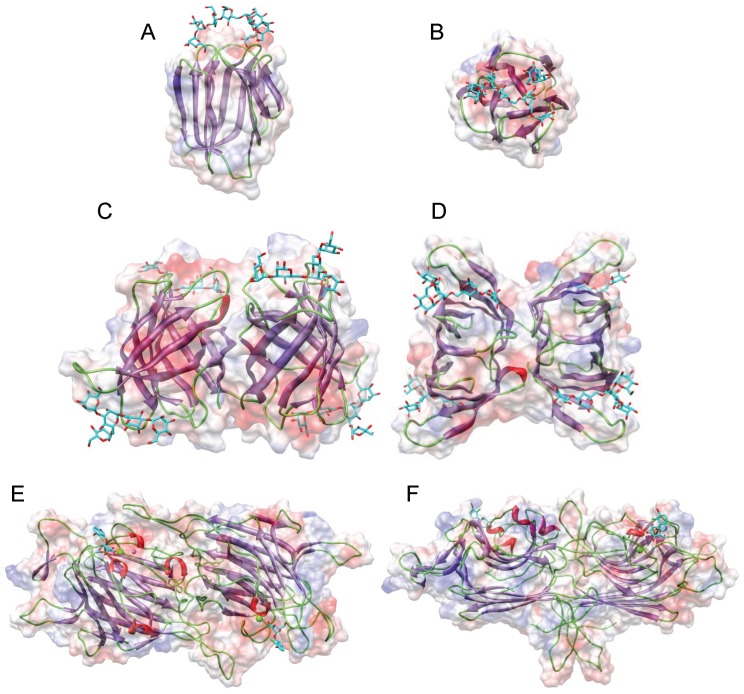
Structural diversity of the mannose-binding seaweed lectins. (**A**,**B**). Lateral (**A**) and front (**B**) views of the ribbon diagram of griffithsin monomer (*protein data bank* (PDB) code 1LL2) in complex with a pentamannoside (colored cyan), showing the β-prism-I organization of strands of β-sheet. Surface electrostatic potential (electronegatively and electropositively charged surfaces colored in red and blue, respectively; neutral surfaces colored grey) is shown in transparency. (**C**,**D**). Lateral (**C**) and front (**D**) views of the ribbon diagram of the modelled *Agardhiella subulata* lectin ASL-1, in complex with a pentamannoside (colored cyan), showing the β-barrel organization of strands of β-sheet. Surface electrostatic potential is shown in transparency. (**E**,**F**). Lateral (**E**) and front (**F**) views of the ribbon diagram of the modelled lectin from *Ostreococcus tauri* OtL, in complex with a dimannoside (colored cyan), showing the β-sandwich organization of strands of β-sheet. Surface electrostatic potential is shown in transparency. ASL-1 and OtL lectins were modelled with YASARA, and rendered with Chimera.

**Figure 2:**
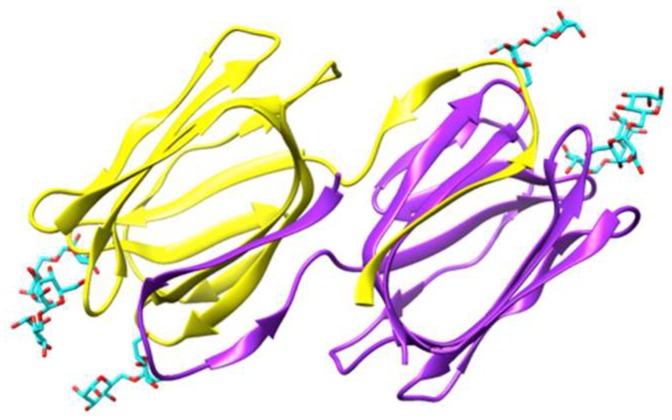
Ribbon diagram showing the domain-swapped structure of griffithsin, organized in two symmetrical domains colored purple and yellow, respectively (PDB code 2HYQ), in complex with six α 1,6-mannobiose ligands (colored cyan). In each domain, the carbohydrate-binding sites (CBS) are located at the top of the β-prism structure. Molecular cartoon drawn with Chimera.

Griffithsin, the man-specific lectin from the red alga *Griffithsia* sp., was among the first man-specific lectins isolated and characterized from seaweeds [3]. The lectin consists of a single polypeptide chain of 122 amino acids which contains three putative *N*-glycosylation sites 46NLS, 72NIS and 105NGS. The X-ray crystal structures of griffithsin revealed a β-prism I organization similar to that found in artocarpin [21] and jacalin [22] from the Jackfruit *Artocarpus integrifolia*, banana lectin [23], and Heltuba from the Jerusalem artichoke *Heliantus tuberosus* [24] (Figure 1A–C). In fact, the lectin consists of a domain-swapped structure resulting from the symmetrical non covalent arrangement of two identical domains (Figure 2). Three carbohydrate-binding sites (CBS) disposed as a triangle at the edges of the β-prism domain, make the two domain-swapped griffithsin an hexavalent man-specific lectin.

### 3.2. Oscillatoria Agardhii Agglutinin Homolog (OAAH) Family

The largest phylum of Rhodophyta contains man-specific lectins which possess the *Oscillatoria agardhii* agglutinin homolog (OAAH) scaffold [25]. The OAAH family comprises the ASL-1 and ASL-2 lectins from *Agardhiella subulata* [8], EDA-2 and ESA-2 from *Eucheuma denticulatum* [10], and *E. serra* [11,12], KAA-2 and KSA-2 from *Kappaphycus alvarezeii* and *K. striatum* [13,14], MEL from Meristiella echninocarpa [8], MPA-1 and MPA-2 from *Meristotheca papulosa* [8], and SfL-1 and SfL-2 .from *Solieria filiformis* [13]. The so-called OAAH scaffold consists of a single polypeptide chain formed by two repeats of five β-strands, organized in a compact 10-stranded β-barrel structure. Two β-barrels associate perpendicularly to build up the complete molecule (Figure 1C,D). This structural scaffold was first identified in the cyanobacterial *Oscillatoria agardhii* lectin (PDB code 3OBL) [26,27], and subsequently found in various red algae lectins. Three CBSs occurring at both ends of the OAAH scaffold make all these man-specific red algae lectins with two OAAH scaffolds, tetravalent lectins.

### 3.3. Legume Lectin-Like Family

**Figure 3:**
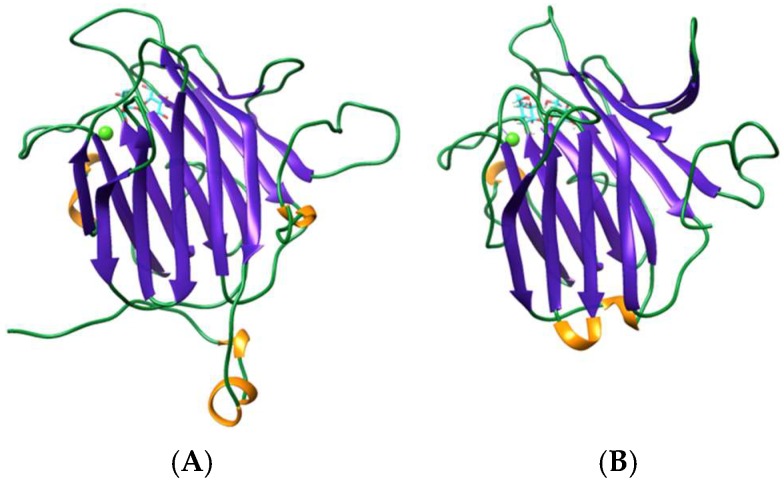
Stereo view showing the conservation of the β-sandwich core between VIP36 protein (PDB code 2DUR) used as a template (**A**), to model the legume-like lectin HFA from *Hydropuntia fisheri* (**B**) in complex with a dimannoside (colored cyan), using YASARA. The Ca^2+^ ion is colored yellow green. The amino acid sequences of proteins share 27.5% identity and 72.5% similarity, and a RMSD between 176 pruned atom pairs and across all 209 atom pairs were 0.908 Å and 2.576 Å, respectively, using the Needleman–Wursch alignment algorithm and the BLOSUM 62 homology matrix. Molecular cartoon drawn with Chimera.

The β-sandwich jelly roll scaffold found in legume lectins (Fabaceae), also occurs in various man-specific lectins from different algae species including the green alga *Ostreococcus tauri* OtL [17], the brown alga *Hydropuntia fisheri* HfL, the yellow brown alga *Nannochloropsis gaditana* NgL [16], and the red alga *Porphyra umbilicalis* which possesses a BU14 protein containing a typical legume-lectin domain [20] (Table 1). The *O. tauri* lectin consists of a homodimer resulting from the non-covalent association of two identical protomers. Each protomer consists of a single polypeptide chain organized in two antiparallel β-sheets of six and seven strands, respectively, to form a β-sandwich structure (Figure 1E,F). This three-dimensional organization is reminiscent of the homodimeric lectin from *Lathyrus nissolia* [28] and *L. sphaericus* [29] in the viciae tribe of Fabaceae. Red algae lectins differ from other homodimeric two-chain lectins of the Viciae tribe, e.g., pea lectin (*Pisum sativum* agglutinin PsA) [30], lentil lectin (*Lens culinaris* agglutinin LcA) [31], yellow vetch lectin (*Lathyrus ochrus* lectin Lol) [32] (Figure 1B), and the faba bean lectin (*Vicia faba* agglutinin VfA or favin) [33], which contain protomers cleaved into a light (α) and a heavy (β) chain. However, like other viciae lectins, they possess two identical mannose-binding sites. They also differ from the single-chain legume lectins, which result from the non-covalent association of four protomers to give homotetrameric mannose-binding lectins occurring in the tribes Baphieae (*Bowringia mildbraedii* agglutinin BMA) [34], Dalbergieae (*Centrolobium tomentosum* lectin CTL [35], *Pterocarpus angolensis* lectin PAL [36]), Diocleae (Con A [37], *Cymbosema roseum* CRL [38], *Dioclea grandiflora* lectin Con GF [39], and other *Dioclea* sp. lectins), which exhibit four mannose-binding sites. Other man-specific lectins like HfL from *Hydropuntia fisheri,* NgL from *Nannochloropsis gaditana* and the legume-lectin domain of the BU14 protein from *Porphyra umbilicalis*, more closely resemble the β-sandwich monomeric structure found in the canine ViP36 lectin (PDB code 2DUR) [40] and the rat p58/ERGIC-53 lectin (PDB code 1GV9) [41], which are similarly built up from two β-sheets of six and seven strands of antiparallel β-sheet associated in a β-sandwich structure (Figure 3). In all of these lectins with a jelly roll scaffold, a single CBS occurs at the confluence of the β-sheets, at the top of the β-sandwich structure. Moreover, in all of these legume-related lectins, some of the amino acid residues forming the CBS also serve to coordinate a Ca^2+^ cation in close proximity, contributing to the conformational stability to the CBS.

### 3.4. GNA-Like Family

**Figure 4:**
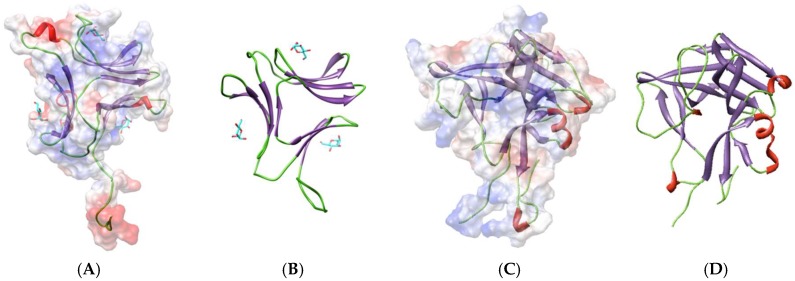
(**A**,**B**) Ribbon diagram of the three-dimensional model built up for the *Boodlea coacta* lectin BCA (**A**), compared to GNA (PDB code 2MSA) (**B**) used as a template for homology modelling (15% identity and 51% similarity, RMSD between 46 atom pairs: 0.457). Surface electrostatic potential (electronegatively and electropositively charged surfaces colored in red and blue, respectively; neutral surfaces colored grey) of BCA are indicated and mannose residues anchored to the CBSs of the lectin are colored cyan. (**C**,**D**) Ribbon diagram of the modelled BPL-2 lectin from *Bryopsis plumosa* (**C**), compared to MFP2 (PDB code 2BJR) (**D**) used as a template for homology modelling (19.5% identity and 48% similarity, RMSD between 82 atom pairs: 0.698 Å). Surface electrostatic potential (electronegatively and electropositively charged surfaces colored in red and blue, respectively; neutral surfaces colored grey) of BCA are indicated. Molecular cartoon drawn with Chimera.

The β-prism-II scaffold, also known as monocot-lectin or GNA-like scaffold, consists of three bundles of four β-strands arranged into a flattened β-prism structure around a central pseudoaxis. A carbohydrate-binding site occurs in a groove located at the center of the bundle of β-strands forming each β-sheet. The man-specific lectin BCA from the brown alga *Boodlea coacta,* [18], exhibits a similar scaffold (Figure 4), and docking experiments performed in silico suggest that both CBSs of BCA are fully active and readily accommodate a mannose residue (see Section 4.4).

### 3.5. MFP2-Like Family

The lectin BPL-2 from the green-yellow alga *Bryopsis plumosa,* has been characterized as a man-specific lectin structurally-related to MFP2, a protein involved in the nematod sperm cell mobility [42]. The lectin consists of three β-harpins which adopt a triangular disposition to form a sort of β-trefoil structure (Figure 4). *In silico* docking experiments performed with mannose, suggest the occurrence of a single active CBS in the BPL-2 lectin (see Section 4.5).

Other man-specific lectins have been isolated and characterized from other alga species, e.g., HLR40-1 and HLR40-2 from the green alga *Halimeda renschii* [16], and EPL-1 and EPL-2 from the green alga *Enteromorpha* (*Ulva*) *prolifera* [43], but none of them resembles the already known seaweed lectin families and thus, no information is yet available on their three-dimensional structures.

**Figure 5:**
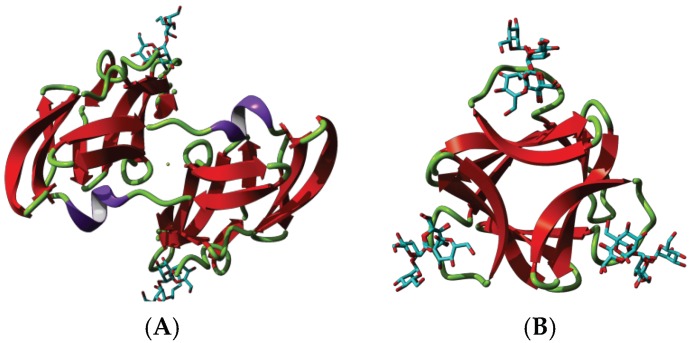
(**A**) Ribbon diagram of the domain-swapped cyanovirin-*N* dimer, in complex with a trimannoside oligosaccharide (cyan colored sticks) at the CBS located at the top of the β-barrel structures (PDB code 3GXY). (**B**) Front view of the ribbon diagram of actinohivin complexed to a tetramannoside oligosaccharide (cyan colored sticks) at the CBS located at the edges of the β-trefoil structure (PDB code 4P6A). Molecular cartoon drawn withYASARA.

Lectins with carbohydrate-binding specificities similar to griffithsin, have been isolated and characterized from bacteria, e.g., cyanovirin-N (CV-N) from the cyanobacterium *Nostoc ellipsosporum* [44], and fungi, e.g., actinohivin (AH) from the Actinomycete *Longispora albida* [45], which both displayed anti-HIV activity [46]. The cyanovirin-N lectin domain consists of a β-barrel (PDB code 2EZM) [47], whereas the actinovirin lectin domain exhibits a β-trefoil architecture (PDB code 3A07) [48]. The accommodation of high-mannose glycans by both lectins, closely resembles that found in griffithsin and other man-specific seaweed lectins, with a prominent role for the oligosaccharide-binding pocket in the CBSs [49,50], (Figure 5).

## 4. Mannose-Binding Specificities of Mannose-Binding Seaweed Lectins

Extensive X-ray crystallographic studies of different lectins complexed to simple and complex carbohydrates revealed the occurrence of two types of closely interlinked carbohydrate-binding specificities at the CBS of plant, algal and fungal lectins [7]:

- A monosaccharide-binding specificity, allowing the lectins to specifically recognize a simple sugar, e.g., mannose, and its derivatives, e.g., α-methylmannoside. This monosaccharide recognition corresponds to the “broad sugar-binding specificity” that allows lectins to accommodate simple sugars in a monosaccharide-binding pocket located within the CBS.
- An oligosaccharide-binding specificity, allowing the lectins to simultaneously accommodate several sugar units of a complex *N*-glycan, e.g., high-mannose glycans, also known as the “fine sugar-binding specificity” of the lectins. This oligosaccharide recognition involves the whole surface of the CBS, including the monosaccharide-binding pocket, which also participates in the binding of the complex glycans.

### 4.1. Griffithsin

The binding of simple sugars (Man, Glc, GlcNAc), dimannoside (α 1,6-mannobiose, maltose) and complex tri-branched high-mannose glycan (Man9GlcNAc2) to griffithsin, has been investigated by X-ray crystallography at atomic resolution (Table 2).

**Figure 6:**
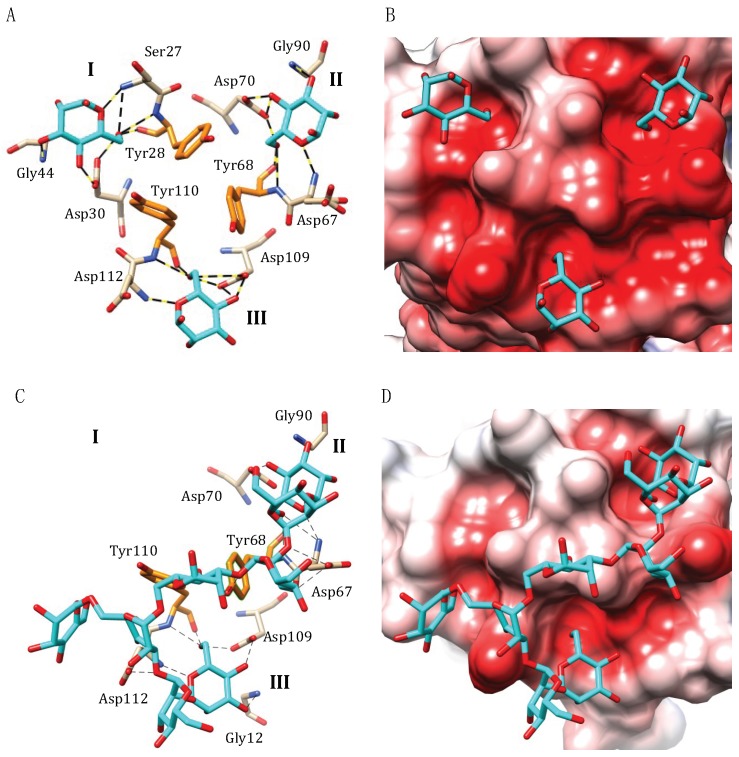
Accommodation of mannose (**A**,**B**) and high-mannose type glycan (**C**,**D**) at the carbohydrate-binding sites of griffithsin (PDB codes 2GUD and 3LL2, respectively). (**A**) Network of hydrogen-bonds (dashed black lines) anchoring mannose to the three CBSs I, II and III, located at the top of monomer A in the dimeric structure of griffithsin (PDB code 2GUD). Stacking interactions occur between tyrosine residues Tyr28 (site I), Tyr68 (site II) and Tyr110 (site III) and the pyranose ring of mannose ligands. Water-mediated H-bonds that participate in the binding of mannose to the CBSs are not represented. (**B**) Coulombic charges (electronegative, electropositive and neutral regions are colored red, blue and white, respectively) at the molecular surface of griffithsin, showing the strong electronegative (acidic) character of the CBDs. (**C**) Network of hydrogen-bonds (dashed black lines) anchoring a high-mannose type glycan to the CBDs of griffthsin (PDB code 3LL2). Note that CBD I does not participate in the binding of the glycan. Stacking interactions occur between tyrosine residues Tyr68 (site II) and Tyr110 (site III) and the pyranose ring of the high-mannose glycan. Water-mediated H-bonds that participate in the binding of high-mannose glycan to the CBDs and the surrounding regions, are not represented. (**D**) Coulombic charges (electronegative, electropositive and neutral regions are colored red, blue and white, respectively) at the molecular surface of griffithsin, showing how the high-mannose glycan chain is accommodated by the strong electronegative (acidic) CBDs and the surrounding regions. Note that the acidic pocket corresponding to CBS I does not participate in the binding of the glycan.

A network of seven hydrogen bonds between O3, O4, O5 and O6 of mannose and Ser27, Tyr28, Asp30 and Gly44 of CBS I, Asp67, Tyr68, Asp70 and Gly90 of CBS II, Asp109, Tyr110 and Asp112 of CBSIII, accommodates the simple sugar in the monosaccharide-binding pocket of the three CBSs located at the top of the griffithsin domain (Figure 6A). Aromatic Tyr28, Tyr68 and Tyr110 residues also participate in stacking interactions with the pyranose ring of the mannose residues, that reinforces the binding of mannose to the CBS. The occurrence of Asp residues in the monosaccharide-binding pocket communicates a strong electronegative character to the griffithsin CBSs (Figure 6B). A very similar binding scheme has been observed in the griffithsin monomer complexed to glucose (PDB code 2NUO) [52], and GlcNAc (PDB codes 2GUE and 2NU5) [51,52].

The binding of disaccharides, α1,6-mannobiose (PDB code 2HYQ) and maltose (PDB code 2HYR) to the griffithsin CBS [53], reveals a very similar binding scheme with a single sugar unit anchored to the monosaccharide-binding pocket via a similar network of hydrogen bonds and stacking interactions with Tyr residues (result not shown). No interaction occurs between the second sugar unit of the disaccharide and the CBS.

The accommodation of more complex sugars by griffithsin, e.g., a tri-antennary high-mannose glycan chain Man9GlcNAc2 (PDB code 3LL2) [54], reveals a multisite interaction between the glycan and CBS II and CBS III of the lectin, the first CBS I remaining vacant and not involved in the interaction with the sugar units of the complex glycan (Figure 6C,D). Only two mannose units located at both ends of the complex high-mannose glycan chain, readily interact with the monosaccharide-binding pocket of CBS II and CBS III via a network of hydrogen bonds and stacking interactions involving the same amino acid residues insuring the anchorage of simple sugars to the CBSs. No contact occurs between other sugar units of the complex glycan and the griffithsin domain (Figure 1A). This multisite binding scheme readily differs from that observed in other plant, algal and fungal lectins, which establish hydrogen bonds and stacking interaction with several sugar units to properly accommodate complex glycans at their extended oligosaccharide-binding site. In this respect, griffithsin uses a similar binding scheme to accommodate both simple sugars, dimannosides and complex high-mannose glycans, which only involves the monosaccharide-binding pockets located at the top of both domains. Finally, the ability to mediate multisite and multivalent interactions with simple sugars accounts for the high affinity and activity of griffithsin toward high-mannose glycans and thus explains the potential anti-viral and anti-cancer activities of the lectin.

### 4.2. OAAH Lectins

**Figure 7:**
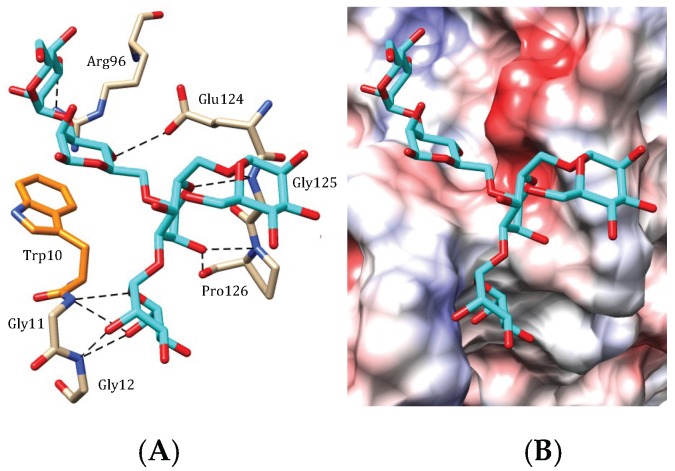
Accommodation of a pentamannosyl glycan (**A**,**B**) at the carbohydrate-binding site of EDA-2, the *Eucheuma denticulata* lectin (modelled and docked, using the PDB code 3OBL as a template). (**A**) Network of hydrogen-bonds (dashed black lines) anchoring the pentamannosyl glycan to the CBS. Stacking interactions occur between tryptophan residue Trp10 and a pyranose ring of the ligand. Water-mediated H-bonds that participate in the binding of pentamannosyl glycan to the CBS are not represented. (**B**) Coulombic charges (electronegative, electropositive and neutral regions are colored red, blue and white, respectively) at the molecular surface of EDA-2, showing the electropositively (blue) and electronegatively (red) charged regions in contact with the pyranose rings of the ligand.

Docking of a two-branched pentamannosyl glycan to the CBS of the modelled EDA-2, the OAAH lectin from the red alga *Eucheuma denticulatum*, reveals an oligosaccharide-binding scheme very similar to those found in plant lectins, especially in legume lectins, involving an extended area around the monosaccharide-binding pocket. A mannose unit anchors to the monosaccharide-binding pocket while other mannose units interact with amino acid residues located in the vicinity of the pocket via a network of hydrogen bonds and a stacking interaction with the aromatic Trp10 residue (Figure 7A). Like in other lectins, the monosaccharide-binding pocket exhibits a strong electronegative (acidic) character (Figure 7B). Docking experiments performed with other modelled OAAH lectins, e.g., the *Solieria filiformis* SfL-1 and SfL-2 lectins, revealed a very similar oligosaccharide-binding scheme at the extended CBS of the lectins.

### 4.3. Legume Lectin-Like Seaweed Lectins

**Figure 8:**
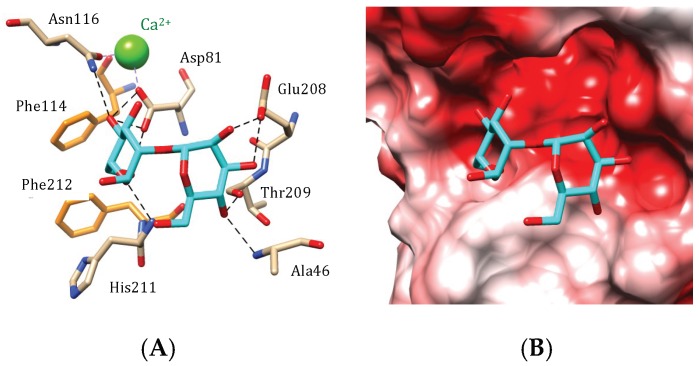
Accommodation of a dimannoside (**A**,**B**) at the carbohydrate-binding site of HFA, the *Hydropuntia fisheri* lectin (modelled and docked, using the PDB code 2DUR as a template). (**A**) Network of hydrogen-bonds (dashed black lines) anchoring the dimannoside to the CBS. Stacking interactions occur between phenylalanine residue Phe114 and Phe212, and a pyranose ring of the ligand. (**B**) Coulombic charges (electronegative, electropositive and neutral regions are colored red, blue and white, respectively) at the molecular surface of HFA, showing the electronegatively (red) charged of the carbohydrate-binding cavity that harbors the sugar.

The accommodation of α1,6-mannobioside to the CBS of the *Hydropuntia* (*Gracilaria*) *fisheri* man-specific lectin (HFA), obeys the oligosaccharide-binding scheme found in legume lectins like Con A and Con A-like lectins [2]. Both mannose units of the disaccharide bind to amino acids located in and close to the monosaccharide-binding site via a network of hydrogen bonds. Additional stacking interactions occurring between the pyranose ring of the mannose units and two aromatic Phe112 and Phe114 residues, complete the interaction of the dimannoside with the CBS (Figure 8A).

Like in other man-specific lectins, the monosaccharide-binding pocket displays a pronounced electronegative (acidic) character (Figure 8B). In addition, like in legume lectins, some of the amino acid residues involved in the H-bond network, e.g., Asp81 and Asn116, also serve as ligands for a Ca^2+^ cation, that reinforces the stability of the CBS and contributes to improve the affinity of the legume lectin-like HFA for mannose and high-mannose glycans.

### 4.4. GNA-Like Seaweed Lectins

**Figure 9:**
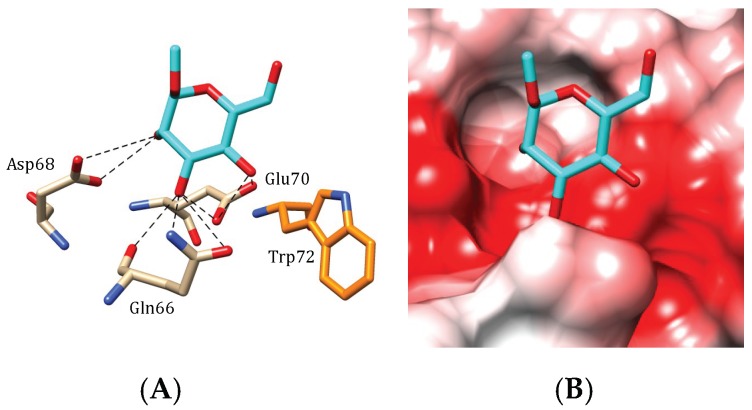
Accommodation of mannose (**A**,**B**) at the carbohydrate-binding site II of BCA, the *Boodlea coacta* lectin (modelled and docked, using the PDB code 1MSA as a template). (**A**) Network of hydrogen-bonds (dashed black lines) anchoring mannose to the CBS. A stacking interaction occurs between tryptophane residue Trp72 and the pyranose ring of the ligand. (**B**) Coulombic charges (electronegative, electropositive and neutral regions are colored red, blue and white, respectively) at the molecular surface of BCA, showing the electronegatively (red) charged character of the carbohydrate-binding cavity that harbors the sugar.

**Figure 10:**
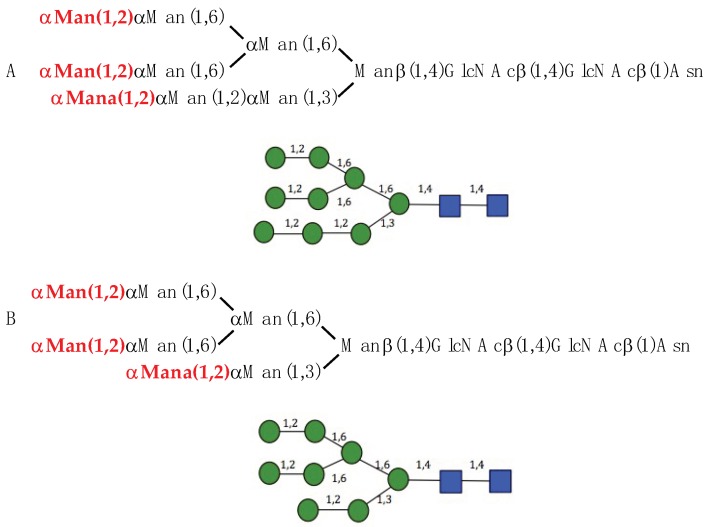
(**A**,**B**) High-mannose glycan chains displaying the higher affinity for BCA. The clusters of terminal α1,2-linked mannose units are shown in red bold letters in both oligosaccharidic structures. Symbols for man (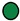) and GlcNAc (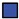) were used to draw the molecular cartoons of high-mannose glycan chains.

The GNA-like seaweed lectin BCA from the brown alga *Boodlea coacta* accommodates mannose at the CBS II via a network of hydrogen bonds linking the O2, O3 and O4 of mannose to residues Gln66, Asp68 and Glu70 forming the monosaccharide-binding pocket of the lectin (Figure 9A). An additional stacking between the pyranose ring of mannose and the aromatic Trp72 residue, complete the interaction. This monosaccharide-binding scheme which also occurs at BBS I and GBS III, strikingly resembles those occurring in the lectins from the monocot mannose-binding or GNA-like lectin family [3]. Calculation of the surface electrostatic potential of BCA indicates a pronounced electronegative (acidic) character of the surface area around the CBSs (Figure 9B), and suggests the capacity for BCA to also accommodate oligomannosidic glycans. In fact, investigations on the oligomannoside-specificity of BCA using a centrifugal ultrafiltration-HPLC technique, have shown that BCA only recognizes high-mannose glycan chains displaying α1,2-linked mannose at the non-reducing terminus [18], which can explain the higher affinity of BCA for α1,2-mannose clusters comprising three terminal α1,2-linked mannose units (Figure 10).

The exclusive specificity for the clusters of terminal α1,2-linked mannose units groups BCA apart from other man-specific seaweed lectins

### 4.5. MFP2B-Like Seaweed Lectins

**Figure 11:**
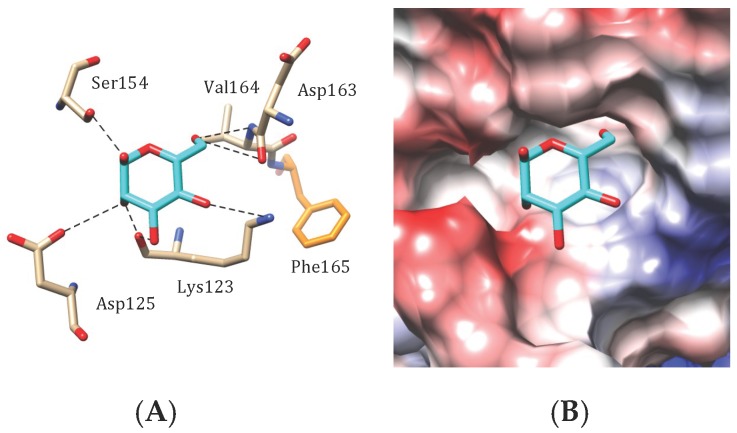
Accommodation of mannose (**A**,**B**) at the carbohydrate-binding site of BPL-2, the *Bryopsis plumosa* lectin (modelled and docked, using the PDB code 2BJR as a template). (**A**) Network of hydrogen-bonds (dashed black lines) anchoring mannose to the CBS. A stacking interaction occurs between phenylalanine residue Phe165 and the pyranose ring of the ligand. (**B**) Coulombic charges (electronegative, electropositive and neutral regions are colored red, blue and white, respectively) at the molecular surface of BPL-2, showing the electronegatively (red) charged character around the carbohydrate-binding cavity that harbors the sugar.

Docking of mannose to the CBS of the modelled BPL-2, the *Bryopsis plumosa* lectin, suggests a monosaccharide-binding scheme very similar to those observed in other man-specific seaweed lectins (Figure 11A). A network of hydrogen bonds between O1, O2, O3, O4 and O6 of mannose and amino acids Lys123, Asp125, Ser154, Asp163 and Val164 forming a monosaccharide-binding pocket, allows the accommodation of the sugar to the CBS of the lectin. An additional stacking interaction occurs between the pyranose ring of mannose and Phe165. The surface around the monosacchaide-binding pocket exhibits an electronegative (acidic) character (Figure 11B).

Finally, the accommodation of high-mannose glycan chains by the man-specific seaweed lectins consists of a complex interaction process in which the monosaccharide-binding pocket plays a prominent role. Similarly, the mannose-binding pocket of the closely-related bacterial lectin cyanovirin-N [49] and fungal lectin actinohivin [50], plays a key role in the accommodation of high-mannose glycans (see Figure 5).

## 5. Phylogenetic Relationships between Mannose-Binding Seaweed Lectins and Higher Plants

**Figure 12:**
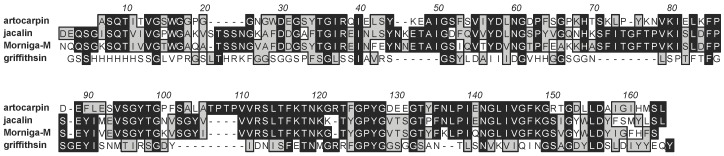
Multiple amino acid sequence alignment of griffithsin and jacalin-related lectins from higher plants (jacalin and artocarpin from *Artocarpus integrifolia*, frutalin from *Artocarpus incisa*, and Morniga-M from *Morus nigra*). Griffithsin shares 25% identity and 70% homology with jacalin-related lectins from higher plants. Sequence alignment was performed with CLUSTAL-X.

The structural resemblance between man-specific lectins from seaweeds and higher plants, raises questions related to the phylogenetic relationships between these man-specific lectins. Compared to the corresponding lectin domains occurring in higher plants, seaweed lectins display amino acid sequences with some degree of conservation, e.g., the comparison between griffithsin and the β-prism I domain found in jacalin-related lectins from higher plants (Figure 12).

**Figure 13:**
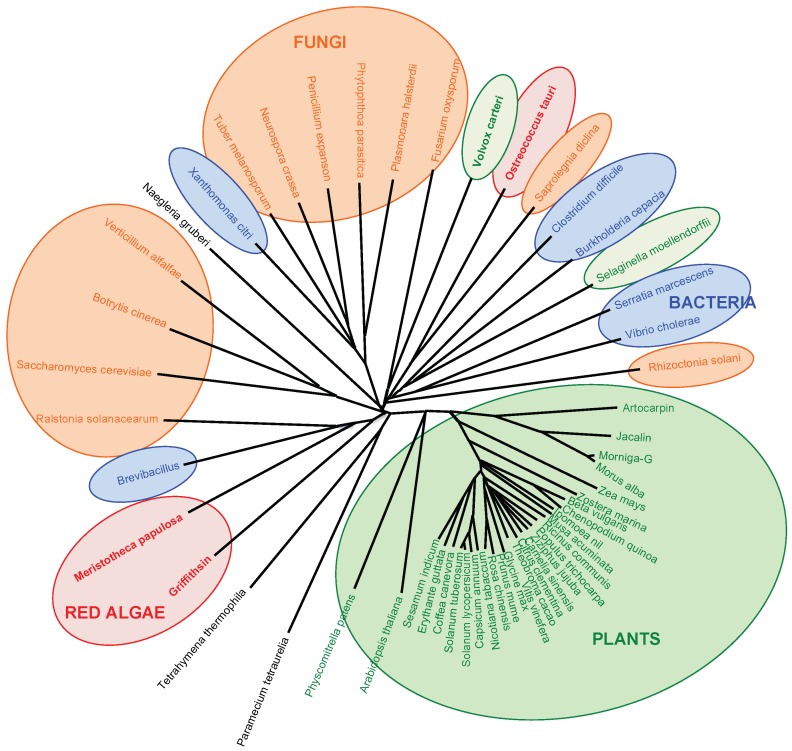
Phylogenetic tree built from the multiple amino acid sequence alignment of jacalin-related lectins from red algae (red boxes), plants (green box), fungi (orange boxes), and bacteria (blue boxes). Lectin of the green alga *Volvox carteri* is green boxed, and lectins from unicellular ciliates are not boxed.

In fact, the jacalin-related β-prism I domain is widespread distributed in all of the living organisms either as an individual lectin, e.g., jacalin or griffithsin, or as a domain associated to other protein components of complex, multidomain proteins from bacteria, fungi and plants, displaying various metabolic functions [20]. In this respect, the man-specific lectins from red algae are more phylogenetically more closely-related to jacalin-related lectins from higher plants, compared to other jacalin-related lectins of fungal or bacterial origin, as shown in the phylogenetic tree built from the multiple amino acid sequence alignment of various jacalin-related lectins from plants, algae, fungi, bacteria and unicellular ciliates (Figure 13). This is not surprising since different genomes of Rhodophyta reveal some close phylogenetic relationships to Viridiplantae [55,56,57,58].

However, depending on the structural scaffold to which they belong, the degree of conservation of the man-specific seaweed lectin domains is extremely variable. Accordingly, the three tandemly arrayed β-prism II domains occurring in BCA, the brown alga *Boodlea coacta* lectin, share a weak degree of conservation compared to other β-prism II domains found in GNA and GNA-like lectins from higher plants, even though all of these lectin domains display a rather well conserved three-dimensional conformation, particularly of the core β-prism structure.

## 6. Biomedical Applications for the Man-Specific Seaweed Lectins

Similar to man-specific lectins from higher plants, biomedical applications of man-specific seaweed lectins were especially applied in two directions, namely their virucidal properties against HIV and their anti-cancer properties.

### 6.1. Mannose-Specific Seaweed Lectins as Virucidal Agents against HIV-I Infection

**Figure 14:**
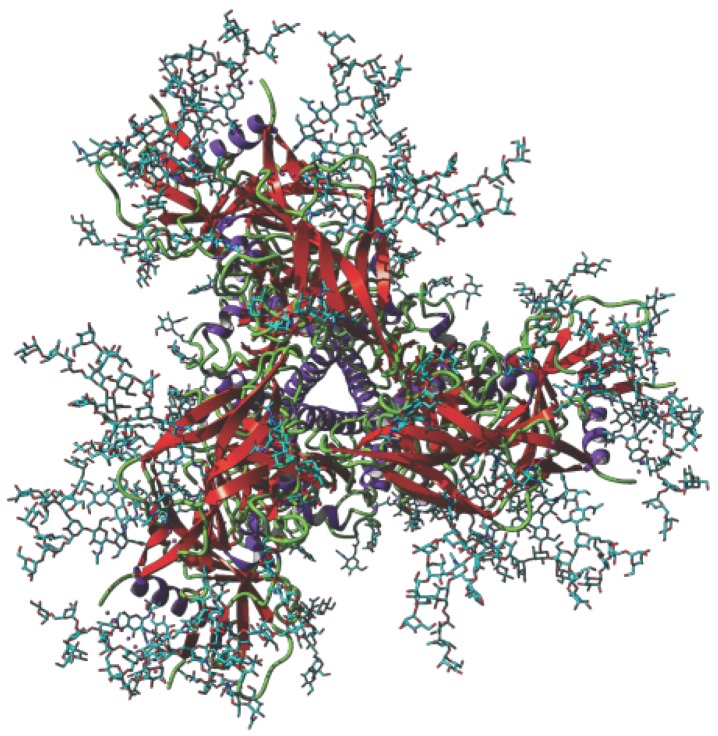
Ribbon diagram of the HIV-1-envelope spike built from the triangular association of three gp120 molecules (PDB code 5FYK). The gp41 molecules which are tandemly associated to the gp120 molecules, are not represented. The fully accessible high-mannose glycan chains that decorate the gp120 units are rendered as cyan colored sticks.

Ever since the high-mannose moiety of *N*-glycans decorating the HIV-1 gp120 protein has been identified as a target recognized by the CD4+ T-lymphocytes in vitro, a step essential to later promote the virion infection of the host cells, man-specific lectins have been deeply investigated as possible agents able to mask the high-mannose target and thus prevent the HIV infection of the host cells by HIV. In fact, the tandem association of three gp120 molecules with three gp41 molecules forms the HIV-1-envelope spike, which consists of a transmembrane trimer of gp41 associated to an extracellular trimer of gp120 exhibiting the exposed high-mannose glycans necessary for the recognition process of HIV-1 by the CD4+ T-lymphocytes [59], (Figure 14). The tandem association of gp120 to gp41 results from the processing of the precursor gp160 by the host cell proteases.

**Figure 15:**
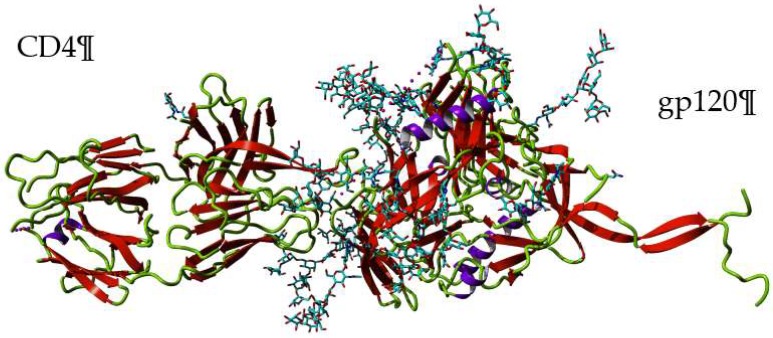
Three-dimensional structure of gp120 complexed to a CD4 molecule (PDB code 5FYK). The high-mannose *N*-glycan chain decorating gp120 are rendered as cyan colored sticks. Lectins which specifically bind to the high-mannose *N*-glycans exposed at the surface of gp120, interfere with the recognition of gp120 by the coreceptors of the CD4+ T lymphocytes.

The specific association of man-specific lectins to the high-mannose glycans of gp120 prevents the subsequent recognition of the HIV-1-envelope spikes by the coreceptors expressing cells, and thus acts as blocking agents for the entry of the HIV-1 virions into the host cells (Figure 15). Accordingly, the removal of two high-mannose *N*-glycans in gp120 resulted in an improved resistance of HIV-1 to griffithsin, the man-specific lectin from the red alga *Griffithsia* sp. [60]. In addition to griffithsin, grifonin-1 (GRFN-1), an 18-mer peptide derived from griffithsin, has also been investigated as a blocking agent against HIV-1 [61]. Moreover, other man-specific seaweed lectins were used as blocking agents for the infection in vitro of CD4+ T-lymphocytes by HIV-1 (Table 3).

The-viral activity of griffithsin depends on its enhanced affinity for the high-mannose glycans decorating gp120 in the HIV-1-envelope spike, even at very low concentrations in the picomolar range. Interaction of griffithsin with gp120 does not prevent the recognition of gp120 by CD4 from the CD4+ T-lymphocytes, but prevents gp120 from interacting with its co-receptors, like e.g., DC-SIGN [64], that inhibits the DC-SIGN-mediated capture and transmission of HIV to CD4+ T-lymphocytes [70]. Due to the multivalent character of the griffithsin, which possesses three CBSs in each monomer of the dimeric structure, multiple interactions occurring between the lectin and the high-mannose glycans of gp120 favor the stability of the gp120-DC-SIGN complex and thus prevent the transmission of HIV to the CD4+ T-lymphocytes [54]. Even though griffithsin is considered an efficient and safe anti-viral agent [63,68,76], the smaller 18-mer peptide derived from griffithsin, grifonin-1 (GRFN-1), should be an excellent substitute since it offers an equivalent anti-viral efficacy associated to a strongly reduced cytotoxicity [61].

The ability of griffithsin to interact in vitro with the exposed mannose residues of the *N*-linked glycans from other envelope viruses, e.g., hepatitis C virus [81,82], herpes virus [71,83], Ebola virus [73], coronavirus [84], and papilloma virus [83], make this carbohydrate-binding agent a potential broad-spectrum tool against various virus infection diseases. In this respect, KAA-2 from the red alga *Kappaphycus alvarezii* [13,80], and ESA-2 from thered alga *Eucheuma serra* [85], were shown to inhibit influenza virus infections by interfering with the virus envelope glycoprotein hemagglutinin.

Other Man-specific lectins of cyanobacterial origin were similarly identified as potent anti-HIV-1 proteins via their specific binding to the envelope gp120, as reported especially for actinohivin [48], cyanovirin-N [47], microvirin from *Microcystis viridis* [86], OAA from *Oscillatoria agardhii* [26], and scytovirin from *Scytonema varium* [66].

### 6.2. Mannose-Specific Lectins as Cancer Biomarkers and Anti-Cancer Drugs

The ability of man-specific plant lectins to discriminate between normal and diseased cancer cells, and specifically recognize the specific changes that have occurred in the high-mannose component covering tumor cells, also exists in man-specific seaweed lectins, allowing these proteins to be used as cytotoxic agents for various malignant cells [5,7]. Accordingly, several man-specific seaweed lectins have been investigated for their cytotoxic activity against various human and mouse cancer cell lines (Table 4).

As reported for plant and fungal lectins [7], the recognition of the altered high-mannose glycans associated to the cancer cells by marine algal lectins, led to programmed cell death through the targeting of different apoptotic and autophagic pathways. At a concentration of 1.2 μg mL^−1^, ESA from *Eucheuma serra* provoked the apoptotic cell death of Colo201, HeLa and MCF-7 cells via the induction of the caspase-3-dependent pathway [90]. The expression of caspase-3 was similarly reported for mouse Colo26 and adenocarcinoma cells, treated in vitro and in vivo conditions by the *E. serra* lectin [91], and for Colo201 cells treated in vitro and in vivo conditions by Span 80 vesicles containing immobilized *E. serra* agglutinin [92]. The *E. serra* lectin ESA, also induced the apoptotic cell death of both murine and human osteosarcoma cells [93]. The cytotoxic effects of SfL from the red alga *Solieria filiformis* on MCF-7, also involve the induction of the apoptotic cell death with an over-expression of caspase-3, -8 and -9 [15].

### 6.3. Other Biomedical Applications of Mannose-Specific Seaweed Lectins

In addition to their anti-viral and anti-cancer properties, miscellaneous biomedical properties of the Man-specific seaweed lectins were investigated, including their anti-bacterial and anti-nociceptive properties, together with their pro-healing effects. An anti-depressant-like effect was also reported in mice for SFL, the man-specific lectin from the red alga *Solieria filiformis*, which likely depends on its interference with the dopaminergic system (Table 5).

## 7. Methods

Pairwise and multiple amino acid sequence comparisons were performed with MacVector, using the CLUSTAL-X program [99]. Hydrophobic cluster analysis (HCA) [100], was performed on HCA plots generated at the RPBS web portal (http://www.mobyle.rpbs.univ-paris-diderot.fr), for the prediction of secondary features along the amino acid sequence of seaweed lectins. An unrooted phylogenetic tree was built up from the multiple alignment of jacalin-related lectins using the uncorrected neighbor joining method integrated in MacVector. No bootstrapping was performed. TreeView 1.6.6 [101], was used to draw the phylogenetic tree of jacalin-related lectins.

Homology modelling of seaweed lectins was performed with the YASARA Structure program [102], using various protein templates from the PDB, depending on the overall structural scaffold to which they belong (e.g., 3OBL of the cyanobacterium *Oscillatoria agardhii* for modelling the *Agardhiella subulata* lectin ASL-1, shown in Figure 1). PROCHECK [103], ANOLEA [104], and the calculated QMEAN scores [105,106], were used to assess the geometric and thermodynamic qualities of the three-dimensional models.

For docking experiments, complex glycans were built using the GLYCAM web server [107], and SWEET II web server [108,109]. The autodockVINA module of YASARA Structure [110], was used to dock the carbohydrate ligand (treated as a flexible molecule) to the lectin model (treated as a rigid molecule). Docking experiments were performed at the SwissDock web server (http://www.swissdock.ch) [111,112], as a control for our docking experiments.

Molecular cartoons were drawn with Chimera [113] and YASARA.

## 8. Discussion

Lectins represent a ubiquitous group of carbohydrate-binding proteins that have been identified in all living organisms [20]. Lectins with various carbohydrate-binding specificities, especially Gal/GalNAc-specific and man-specific lectins, have been characterized in all groups of algae, primarily in green algae or Chlorophyta, brown algae or Ochrophyta, and red algae or Rhodophyta [46]. Except for a few lectins built up from structural scaffolds with no apparent homology with those found in the different classes of plant lectins [114], many other seaweed lectins share structural scaffolds in common with higher plant lectins, e.g., the jacalin-related scaffold, the legume-lectin scaffold and the GNA-like scaffold, irrespective of their carbohydrate-binding specificity. Likely, these structural homologies reflect the phylogenetic relationships that have occurred between higher plants and seaweeds during evolution [20].

Mannose-specific seaweed lectins occur in different phyla of algae but red algae or Rhodophyta, are particularly rich in species containing man-specific lectins [115]. To date, much attention has been paid to red algae lectins because of their potential biomedical properties [5]. In this respect, griffithsin, a lectin with a strong affinity for high-mannose glycans, isolated from the red alga *Griffithsia* sp. [3], was extensively investigated due to its enhanced anti-viral properties against HIV-1 and other pathogenic enveloped viruses [5]. Compared to other bacterial and plant lectins used as HIV entry inhibitors, griffithsin was reported as an efficient drug, at extremely low concentrations in the picomolar range, and safer toward uninfected cells [63,68,76]. In addition, the smaller substitute, an 18-mer peptide derived from griffithsin, grifonin-1 (GRFN-1), offers an equivalent efficacy associated to an improved safety toward healthy cells [61].

Besides their anti-viral properties, man-specific seaweed lectins also display relevant cytotoxic properties against various cancer cells by virtue of their capacity to target specific changes that have occurred in the high-mannose glycans expressed at the cell surface of cancer cells [116,117,118,119,120,121,122,123,124,125,126,127,128].

In spite of their biomedical potentialities as anti-HIV and anti-cancer drugs, only very few biomedical applications have been initiated with Man-specific seaweed lectins, essentially because of the difficulties inherently associated to the large-scale production of natural products. Even though scalable manufacture has been proposed for griffithsin [63], and high yields of production were previously reported for the isolation of *Euchema* lectins [9], the large scale production of algal lectins still remains a difficult task associated with unprofitable industrial yields. Additionally, a few topical administrations of griffithsin in combination with Span 80 vesicles [92], and carrageenan [83,129], applications based on the use griffithsin to prevent different enveloped virus infections are rare. Similarly, the synergistic association of griffithsin to the tenofovir, maraviroc and enfuvirtide drugs [64], and other carbohydrate-binding agents (CBAs) like the monoclonal antibody 2G12 and microvirin (MVN) [130], have been proposed in topical microbicide applications. Previously, the *Eucheuma serra* lectin ESA, had been successfully immobilized on the surface of lipid vesicles and the resulting ESA-bearing lipid vesicles were shown to effectively bind to cancer cell lines Colo201 and HeLa [90]. Delivery of griffithsin from griffithsin-modified electrospun fibers was successfully tested as an efficient and safe delivery scaffold for preventing HIV infection [131]. Recently, the monoclonal antibody 2G12 was simultaneously produced in rice endosperm with griffithsin and cyanovirin-N and, unexpectedly, extracts of transgenic plants expressing both proteins were shown to display an enhanced in vitro binding to gp120 and synergistic HIV-1 neutralization [132].

## Figures and Tables

**Table 1:** Overview of algal lectins with a mannose-specificity. OAAH— *Oscillatoria agardhii* agglutinin homolog; GNA—Galanthus nivalis agglutinin.

Algae Phylum	Algae Family	Algae Species	Lectin	Structural Scaffold	Ref.
Red algae	Griffithsin	*Griffithsia* sp.	griffithsin	β-prism I	[3]
Brown algae	OAAH-like	*Agardhiella subulata*	ASL-1,	β-barrel	[8]
			ASL-2	β-barrel	
		*Eucheuma amakusaensis*	EAA-1	β-barrel	[9]
			EAA-2	β-barrel	
			EAA-3	β-barrel	
		*Eucheuma cottonii*	ECA-1	β-barrel	[9]
			ECA-2	β-barrel	
		*Eucheuma denticulatum*	EDA-1	β-barrel	[10]
			EDA-2	β-barrel	
		*Eucheuma serra*	ESA-1	β-barrel	[11,12]
			ESA-2	β-barrel	
		*Kappaphycus alvarezii*	KAA-2	β-barrel	[13]
		*Kappaphycus striatum*	KSA-2	β-barrel	[14]
		*Meristiella echinocarpa*	MEL	β-barrel	[8]
		*Meristotheca papulosa*	MPA-1	β-barrel	[8]
			MPA-2	β-barrel	
		*Solieria filiformis*	SfL-1	β-barrel	[15]
			SfL-2	β-barrel	
Yellow-	Legume-like	*Hydropuntia fisheri*	HFA	β-sandwich	[Ac. GQ906709]
		*Nannochloropsis gaditana*	NgL	β-sandwich	[16]
		*Porphyra umbilicalis*	BU14	β-sandwich	[Ac. OSX69288]
		*Ostreococcus tauri*	OtL	β-sandwich	[17]
green algae	GNA-like	*Boodlea coacta*	BCA	β-prism II	[18]
Green algae	MFP2-like	*Bryopsis plumosa*	BPL-2	MFP2-like	[Ac. BAI43482]
				scaffold?	
	unknown	*Halimeda renschii*	HRL40-1/2	β-prism I	[19]

**Table 2:** PDB codes of lectins from seaweeds, complexed to mannose, glucose, *N*-acetyl-glucosamine, maltose and high-mannose glycans.

Algae Species	Lectin	PDB Code (Complexed Sugar)	Ref.
*Griffithsia* sp.	griffithsin	2GUC, 2GUD (Man)	[51]
		2NUO (Glc)	[52]
		2GUE (GlcNAc), 2NU5 (GlcNAc)	[51,52]
		2HYQ (α 1,6-mannobiose), 2HYR (maltose)	[53]
		3LL2 (9ManGlcNAc2)	[54]

**Table 3:** List of the man-specific seaweed lectins inhibiting HIV infection by binding to the viral gp120 envelope protein. EC_50_ is in the range between 0.1 nM and 1.8 nM, depending on differences in methods used to quantify the anti-HIV activity of griffithsin in vitro. IC_50_ is usually less than 1.0 nM.

Algae Phylum	Algae Family	Lectin	Ref.
Red algae	Griffithsin	Griffithsin (*Griffithsia* sp.)	[4,54,61,62,63,64,65,66,67,68,69,70,71,72,73,74,75,76,77,78,79]
(Rhodophyta)		GRFN-1 or Grifonin-1 (*Griffithsia* sp.)	[61]
	OAAH-like family	KAA-2 (*Kappaphycus alvarezii*)	[80]
Green algae	GNA-like family	BCA (*Boodlea coacta*)	[4,18]
(Chlorophyta)	Legume-like family	OtL (*Ostreococcus tauri*)	[17]

**Table 4:** Cytotoxic effects of Man-specific lectins on cancer cells (reported during the last decade). Apopt.: apoptosis.

Phylum	Species	Lectin	Cancer cell	Apopt.	Ref.
Red algae	*Agardhiella tenera*	ATA	mouse leukemia cell L5178Y		[87]
	*Bryoyhamnion seaforthii*	BSL	human colon carcinoma cells L5178Y		[88]
			oligodendroglioma, ependymona,		[89]
			meningioma, medullo-blastoma		
	*Bryothamnion triquetrum*	BTL	human colon carcinoma	+	[89]
	*Eucheuma serra*	ESA	Colo201, HeLa	+	[90]
	*Solieria filiformis*	SfL-1	mouse Colon26 adenocarcinoma	+	[91]
		SfL-2	Colo201	+	[92]
			human osteocarcinoma, murine	+	[93]
			osteocarcinoma LM8		
			MCF-7		[15]

**Table 5:** Other investigated biomedical properties of Man-specific seaweed lectins.

Alga Phylum	Alga Species	Lectin	Biomedical Property	Ref.
Red algae	*Eucheuma serra*	ESA	Anti-bacterial	[94]
	*Solieria filiformis*	SfL	Anti-bacterial	[95]
	*Solieria filiformis*	SfL	Anti-nociceptive	[96]
	*Solieria filiformis*	SfL	Anti-depressant	[97]
	*Bryothamnion seaforthii*	BSL	Pro-healing	[98]

## Abbreviations

CBA	Carbohydrate-binding agent
CBM	Carbohydrate-binding module
CBS	Carbohydrate-binding site
Con A	Concanavalin A
CVN	Cyanovirin N
GNA	Galanthus nivalis agglutinin
Heltuba	Helianthus tuberosus agglutinin
HIV	Human Immunodeficiency virus
LCA	Lens culinaris agglutinin
OAA	Oscillatoria agardhii agglutinin
PDB	*Protein data bank*
PsA	*Pisum sativum agglutinin*
VfA	*Vicia faba agglutinin*
